# Validation of the Korean Academy of Geriatric Dentistry screening questionnaire and oral frailty diagnostic criteria in community-dwelling older adults

**DOI:** 10.4178/epih.e2024008

**Published:** 2023-12-11

**Authors:** Jeong-Hyun Kang, Seong-Chan Park, Hoi-In Jung, Sun Jae Jung, Hye-Jin Park, Soo-Min Kim, Min-Ji Jo, Yun-Seon Lee, Sun-Young Han

**Affiliations:** 1 Clinic of Oral Medicine and Orofacial Pain, Institute of Oral Health Science, Ajou University School of Medicine, Suwon, Korea; 2 Oral Science Laboratory, Department of Dental Hygiene, College of Software and Digital Healthcare Convergence, Yonsei University, Wonju, Korea; 3 Department of Preventive Dentistry & Public Oral Health, Yonsei University College of Dentistry, Seoul, Korea; 4 Department of Preventive Medicine, Yonsei University College of Medicine, Seoul, Korea; 5 Department of Public Health, Yonsei University Graduate School, Seoul, Korea

**Keywords:** Aging, Frailty, Oral frailty, Diagnosis

## Abstract

**OBJECTIVES:**

This study aimed to establish the validity—specifically, the sensitivity and specificity—of the screening questionnaire and diagnostic criteria for oral frailty proposed by the Korean Academy of Geriatric Dentistry (KAGD) among community-dwelling older adults.

**METHODS:**

This study enrolled 100 participants. Among various definitions of oral frailty, this study used the criteria proposed by Tanaka as the reference test. The screening questionnaire consisted of 11 items for screening physical frailty, chewing ability, swallowing difficulties, oral dryness, and tongue and lip motor function. Each question had a different scoring weight, and if the total score was 1 or higher, an oral frailty diagnostic examination proposed by the KAGD would be recommended. The diagnostic test was the oral frailty diagnostic criteria proposed by the KAGD including 6 measures: chewing ability, occlusal force, tongue pressure, oral dryness, swallowing difficulty, and oral hygiene. If a participant exhibited 2 or more positive measures, this participant was classified as “oral frail.” The screening questionnaire was analyzed using a cut-off value of 1 or higher, while the diagnostic criteria utilized a cut-off of 2 or more positive measures. Sensitivity and specificity were calculated.

**RESULTS:**

The screening questionnaire showed significant power for screening oral frailty (area under the receiver operating characteristic curve, 0.783; sensitivity, 87.8%; specificity, 52.5%). The diagnostic accuracy of the newly proposed diagnostic criteria was acceptable (sensitivity, 95.1%; specificity, 42.4%).

**CONCLUSIONS:**

The newly proposed screening questionnaire and diagnostic criteria in Korea appear to be a useful tool to identify oral frailty in community-dwelling older adults.

## GRAPHICAL ABSTRACT

[Fig f4-epih-46-e2024008]

## Key Message

This study aimed to validate a screening questionnaire and diagnostic criteria for oral frailty among older adults living in the community. Sensitivity and specificity were calculated, with the screening questionnaire demonstrating a significant power for identifying oral frailty (sensitivity 87.8%, specificity 52.5%) and the diagnostic criteria showing acceptable accuracy (sensitivity 95.1%, specificity 42.4%). The findings suggest that the proposed screening questionnaire and diagnostic criteria are valuable tools for identifying oral frailty in community-dwelling older adults in Korea.

## INTRODUCTION

The potential impacts of deteriorating oral health conditions and function on the onset and progression of frailty, disability, and mortality have been previously proposed [1-8]. To adequately assess and address the oral well-being of the elderly population, it is essential to establish clear concepts and reliable diagnostic criteria for geriatric oral dysfunction. In pursuit of this goal, several tools and indices have been developed to evaluate the presence and extent of geriatric dysfunction in the oral and maxillofacial regions [8-12]. The concept of oral frailty has also been introduced [8,10,13]. In 2010, Japan became a super-aged society and recognized the importance of oral care for the elderly. In response to the demographic shift, the Japanese Society of Gerodontology issued a position paper that outlined the diagnostic criteria for oral hypofunction [10]. This led to the official recognition of deteriorated oral health and function as a disease in Japan.

Frailty is defined as an age-related decline in physiological reserves and function across multiple organ systems, making individuals more susceptible to adverse health outcomes [14]. The rising prevalence of frail, dependent older adults poses a significant challenge in aging societies, due to the increased social and medical costs associated with their care. In contemporary times, changes in social structures and the growing medical needs that accompany longer life expectancies and a burgeoning elderly population are global trends. Consequently, within an aging society, early identification of risk factors for frailty and implementation of suitable interventions to prevent older adults from becoming dependent and frail older adults are important issues for both clinicians and researchers.

Korea is facing one of the most rapid shifts toward an aging society due to its low birth rate and a substantial elderly population, pushing it toward becoming a super-aged society. This demographic change has made the development of effective tools for detecting and managing risk factors associated with disability and frailty a top priority. The Korean Academy of Geriatric Dentistry (KAGD) has introduced a clinical guideline, a screening questionnaire, and diagnostic criteria to identify oral frailty [12,15]. These instruments were crafted using evidence-based methods, with a focus on clinical relevance and cultural considerations. They include clinical recommendations, screening questionnaires, and diagnostic criteria for oral frailty. However, the accuracy of the proposed diagnostic criteria and screening tool has not yet been validated. In the field of geriatric medicine and dentistry, Tanaka’s criteria are widely favored for diagnosing oral frailty [15]. Therefore, this study aimed to determine the validity of the KAGD screening questionnaire and diagnostic criteria for oral frailty, as measured by their sensitivity and specificity, within community-dwelling older adults.

## MATERIALS AND METHODS

### Participants

This study was conducted according to the Standards for Reporting Diagnostic Accuracy Studies (STARD) [16].

**Figure 1.**
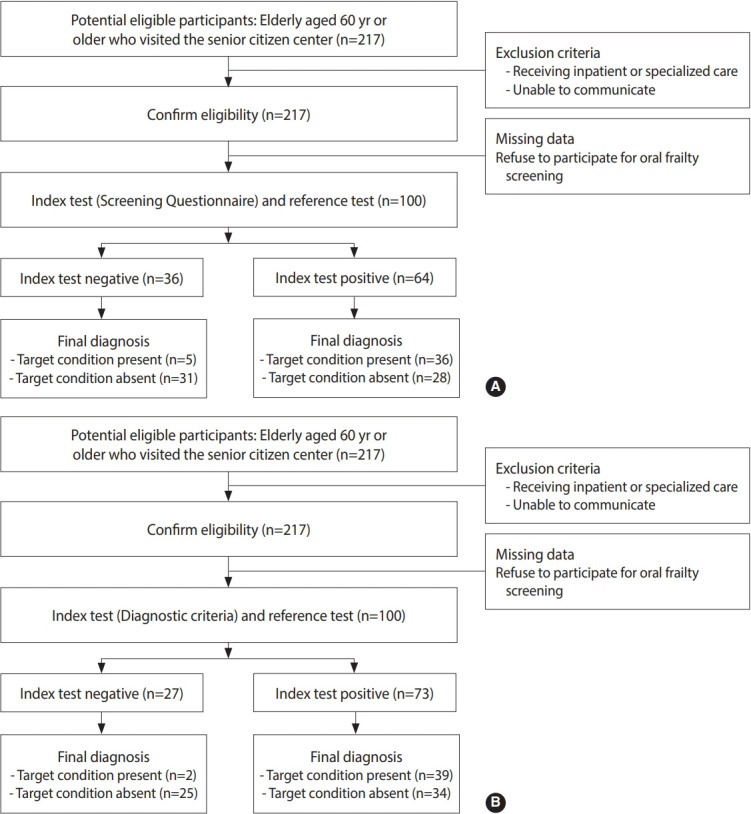
Results determined using the (A) screening questionnaire and (B) diagnostic criteria as an index test.

To recruit participants for the study, we proactively reached out to senior centers in Wonju, Gangwon Province, focusing on areas with a high density of elderly residents. One of the authors (SCP) visited 17 locations where the senior centers had granted permission. Initially, 217 elderly individuals, all over the age of 60, who were visiting the centers voluntarily, were recruited. We excluded participants who had recently received inpatient or specialized care, or those who were unable to complete surveys and oral examinations due to impaired consciousness or communication difficulties. Out of the 217 participants, 100 provided consent and successfully completed both the screening questionnaire and the oral frailty diagnostic examination (Figure 1).

All participants signed an informed consent form. A single author (SCP) interviewed all the participants and gathered information regarding their underlying diseases and current and past medication history. Information on systemic diseases was collected using open-ended questions, such as “Have you been diagnosed with any medical conditions by a specialist? If so, what is the condition?” The oral examination items were measured by trained examiners (HJP, SMK, MJJ, YSL).

### Reference standard

Oral frailty was diagnosed using the criteria proposed by Tanaka, which serve as a reference and include 6 measures. These measures are: (1) the number of present natural teeth, (2) chewing ability assessed with color-changing chewing gum, (3) articulatory oral motor skills evaluated through oral diadochokinesis, (4) tongue pressure, and subjective difficulties with, (5) eating, and (6) swallowing [8]. A participant was classified as “oral frail” if they exhibited more than 3 or more positive measures out of the 6.

The number of natural teeth present in the oral cavity was assessed by a single author (SCP). Chewing ability was evaluated using a color-changing chewing gum (XYLITOL; Lotte, Tokyo, Japan). Participants were instructed to chew the gum for 1 minute in a manner similar to how they would chew food, and the resulting color change of the gum (from green to red) was observed. A chewing disability was identified when the evaluation of the gum’s red photogenesis yielded a Δa value ≤ 10.8.

Articulatory oral motor skill disability was assessed using oral diadochokinesis, specifically the syllables [pʌ], [tʌ], and [kʌ] [17]. Participants were instructed to repeat each syllable as rapidly as they could for a duration of 5 seconds. An oral motor skill disability was identified if the production rate of the syllable [tʌ] was less than 5.2 repetitions per second for elderly men and less than 5.4 repetitions per second for elderly women.

Tongue pressure was measured using a hand-held balloon probe and manometer (JMS tongue pressure measuring instrument TPM-02; JMS Co., Ltd, Tokyo, Japan) [18]. Participants were instructed to compress the balloon attached to the probe against their anterior palate by exerting their tongue’s maximum voluntary force. Decreased tongue pressure was diagnosed when the maximum tongue pressure was less than 27.4 kPa for elderly men and less than 26.5 kPa for elderly women.

Two subjective questions from the Kihon Checklist were used regarding eating and swallowing difficulties: “Do you experience any difficulties eating tough foods compared with 6 months ago?” and “Have you choked on your tea or soup recently?” [19]. Based on these 6 measures, a participant was diagnosed with oral frailty if they exhibited more than 3 or more positive measures.

### Index tests

In the present study, we aimed to assess the validity of both the self-administered oral frailty questionnaire and the oral frailty diagnostic criteria developed by the KAGD as index tests in relation to the oral frailty criteria proposed by Tanaka.

#### Screening questionnaire

The screening questionnaire consisted of 11 items for screening physical frailty, chewing ability, swallowing difficulties, oral dryness, and tongue and lip motor function (Supplementary Material 1; range, 0-15). Each question had a different scoring weight, and if the total score was 1 or higher, an oral frailty diagnostic examination would be recommended (Supplementary Material 2). The response results were aggregated and categorized into high-risk (3.5 to 15.0 points), risk (1.0 to 3.0 points), and normal (0.0 to 0.5 points) groups.

#### Diagnostic criteria of oral frailty proposed by the KAGD

The index test was the oral frailty diagnostic criteria proposed by KAGD. The criteria included 6 measures: chewing ability, occlusal force, tongue pressure, oral dryness, swallowing difficulty, and oral hygiene [12]. If a participant exhibited 2 or more positive measures among these 6 measures, this participant was classified as having “oral frail”. Participants with 1 positive measure were regarded as having “pre-oral frail” status, whereas those exhibiting no positive criteria were classified as having a “robust” status (Supplementary Material 2).

Chewing ability was assessed using a color-changing chewing gum (XYLITOL; Lotte) as in Tanaka’s method. The evaluation was primarily conducted using a specific color scale provided by the manufacturer. In cases where the gum’s color fell between two criteria, the assessment was made in favor of the higher-risk category. The researchers responsible for judging were trained to ensure consistent evaluations, achieving a Fleiss kappa value of 0.778 (p< 0.001). Then participants were grouped into those with chewing disability (color level 1 or 2) and those without (color level 3-5), depending on the color observed.

The maximum occlusal force was assessed by having participants bite down on a pressure-sensitive film (Dental Pre-scale II; CF Co., Ltd, Tokyo, Japan) in the maximum intercuspal position for 3 seconds. The resulting data were analyzed using a dedicated scanner (GT-X830; Epson, Tokyo, Japan) and corresponding software (Bite Force Analyzer; GC Co., Ltd., Tokyo, Japan). Participants were instructed to clench the film with their maximum bite force twice. An occlusal force of less than 500 N was considered reduced.

Tongue pressure was measured using a tongue pressure measuring instrument (JMS tongue pressure measuring instrument TPM-02; JMS Co., Ltd.). The methodology and participant instructions adhered to the protocol established by Tanaka. Each participant’s tongue pressure was measured three times, and the highest value obtained was utilized for evaluation. A maximum tongue pressure below 30 kPa was indicative of decreased tongue pressure.

An oral moisture checker (Mucus; Life Co., Ltd., Saimata, Japan) was used to assess mucosal wetness on the dorsal surface of the tongue. A participant was diagnosed with oral dryness if the mean of 3 measurements taken with the device was below 27.9.

Difficulty swallowing was determined using the modified water swallowing test [20]. Participants received 3 mL of cold water in their oral vestibule and were asked to swallow it. If they could swallow the water without choking, exhibiting wet hoarseness, or experiencing changes in their breathing, they were allowed up to two more attempts to confirm the findings. The poorest result from these attempts was then recorded as the participant’s final assessment. Should choking, wet hoarseness, or changes in breathing have occurred, the participant was considered to have swallowing difficulty. In the scoring of the modified swallowing test, a score of 3 or lower indicated swallowing difficulty. Alternatively, a food test may be employed to assess swallowing difficulty [20].

The Oral Health Assessment Tool (OHAT), specifically the section on oral cleanliness, was employed to evaluate participants’ oral hygiene status [21]. An OHAT score of 1 indicated that the participant’s mouth or dentures were free of food particles and tartar. A score of 2 was assigned if food particles, tartar, or plaque were present in 1-2 areas of the mouth or dentures, or if the participant had halitosis. A score of 3 was given when food particles, tartar, or plaque were found in most areas of the mouth or dentures, accompanied by severe halitosis. Participants with an OHAT score of 2 or higher were considered to have poor oral hygiene status.

### Statistical analysis

The diagnostic accuracy, as indicated by sensitivity and specificity, was assessed by comparing the results from the screening questionnaire and the diagnostic criteria by KAGD against the reference standard (Tanaka’s diagnostic criteria for oral frailty). For the screening questionnaire, a cut-off value of 1 or higher was utilized, while the diagnostic criteria required more than 2 positive measures, following the methodology of a previous study [12]. Participants were categorized based on whether they received a positive or negative diagnosis of oral frailty. In addition to sensitivity and specificity, other statistical measures such as the positive likelihood ratio (LR+), negative likelihood ratio (LR-), positive predictive value (PPV), and negative predictive value (NPV) were computed based on this study’s results. A receiver operating characteristic (ROC) curve was constructed, and the area under the curve (AUC) was determined to evaluate the efficacy of the screening questionnaire in diagnosing oral frailty. Data analyses were conducted using the software Stata version 18.0 (StataCorp., College Station, TX, USA).

### Ethics statement

The research protocol was approved by the Institutional Review Board (1041849-202212-SB-239-02) of Yonsei University Mirae Institutional Review Board. All participants provided written informed consent.

## RESULTS

### Demographic features, oral health status, and oral function of the participants

Of the 217 participants, 100 met the inclusion criteria and provided informed consent (Figure 1). During participant recruitment, we excluded older adults who were unable to communicate effectively. No individuals with dementia visited the senior center throughout the duration of the study. Furthermore, all participants were sufficiently healthy to sign their names and complete the questionnaire. The participants were then categorized into three groups according to the oral frailty diagnosis criteria proposed by KAGD, equivalent to “robust,” “pre-frail,” and “oral frail,” with 7 participants, 20 participants, and 73 participants in each group, respectively. The oral frailty group consisted of significantly older individuals and a higher proportion of females compared to the other groups (Table 1). A total of 12 systemic diseases were identified through self-reporting, and the presence or absence of these diseases is detailed in Table 1. Notably, no participants reported diseases related to oral function, such as oral cancer, or dementia that could interfere with study participation. Participants with oral frailty had a significantly lower number of remaining teeth (p< 0.001), reduced chewing ability (p= 0.049), occlusal force (p< 0.001), and tongue pressure (p< 0.001), as well as a higher prevalence of oral dryness (p< 0.001). Conversely, swallowing function, oral hygiene, and oral diadochokinetic ability did not exhibit significant differences in relation to the degree of the oral frailty (Table 2).

### Validity of the screening questionnaire and diagnostic criteria proposed by the Korean Academy of Geriatric Dentistry

**Figure 2.**
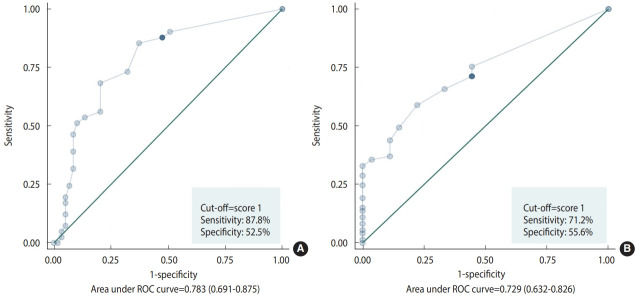
Receiver operating characteristic (ROC) curve of the screening questionnaire for screening oral frailty (cut-off ≥1, high sensitivity and low specificity) based on (A) Tanaka’s criteria, (B) the criteria proposed by the Korean Academy of Geriatric Dentistry.

Thirty-six percent of the participants had no positive responses, while the remaining 64% scored 1.0 or higher on the oral frailty screening questionnaires. The questionnaire demonstrated considerable efficacy in screening for oral frailty, with a sensitivity of 87.8%. The diagnostic accuracy of the proposed criteria for oral frailty was deemed acceptable, with a sensitivity of 95.1% and a specificity of 42.4%, as shown in Table 3 and Figure 2A.

The ROC curve analysis revealed that a cut-off value of for the oral frailty screening questionnaire yielded acceptable diagnostic accuracy, with a sensitivity of 71.2% and a specificity of 55.6% (AUC= 0.729, p< 0.001; Figure 2B). This cut-off value aligns with that proposed in a previous report, suggesting that the screening questionnaire may be suitable for use in dental clinic settings.

### Correlation between results from oral frailty screening questionnaire and diagnostic criteria proposed by the Korean Academy of Geriatric Dentistry

**Figure 3.**
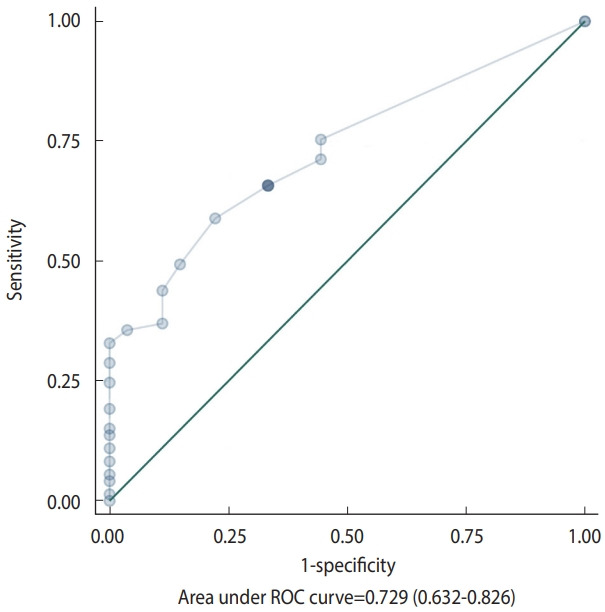
Receiver operating characteristic (ROC) curve of the screening questionnaire proposed by the Korean Academy of Geriatric Dentistry to predict oral frailty (cut-off ≥2, optimal sensitivity and specificity).

Significant correlations were detected between the scores from the oral frailty screening questionnaire and the results from the proposed oral frailty diagnostic criteria (Table 4). The ROC curve analysis revealed that the screening questionnaire demonstrated acceptable diagnostic accuracy for the newly proposed criteria, with an optimal cut-off value of 2, based on a sensitivity of 58.9% and a specificity of 77.8% (AUC= 0.729, p< 0.001) (Figure 3).

## DISCUSSION

The increasing population of dependent and frail older adults poses a significant challenge in an aged society. Therefore, early detection and effective management of frailty risk factors are crucial. Poor oral health has been identified as a significant risk factor that contributes to the development and progression of frailty [1-8]. The concept of “oral frailty” has emerged, with various diagnostic criteria and screening tools designed to assess the oral health and function of older adults [8-12]. Consequently, this study aimed to evaluate the validity of a new screening questionnaire and diagnostic criteria for oral frailty, as proposed by KAGD, in community-dwelling older populations.

The relationship between oral health and the incidence and progression of frailty is well-established, prompting the inclusion of oral health criteria in several diagnostic tools and clinical guidelines for frailty [22-24]. Typically, these criteria, indices, or guidelines have relied heavily on questions posed by clinicians to patients during clinical visits or on self-reported questionnaires. While subjective satisfaction with oral health and function is important, objective measures are crucial for accurately assessing and tracking the current condition and its progression. Notably, the results concerning subjective satisfaction with eating and swallowing did not correlate significantly with objective measures, including the diagnostic criteria proposed in Korea. A previous study found that objective mixing ability was significantly associated with the onset of frailty, whereas self-perceived masticatory difficulty was not [25]. In older adults, who may have compromised cognitive function, relying solely on subjective measures could lead to skewed results. Consequently, valid, reproducible, and objective evaluations of oral function and health are essential for the proper management of frailty.

However, assessing oral frailty can be challenging due to the specialized training and equipment required for dental professionals. A screening questionnaire has been proposed as a more accessible alternative for detecting early signs of deteriorating oral health and function. This tool can be used by caregivers or guardians, even if they are not dental professionals. The newly proposed screening questionnaire includes 11 items that address various aspects of oral and general health, such as physical frailty, chewing ability, swallowing difficulties, oral dryness, and the motor function of the tongue and lips. With a cut-off value of ≥ 1, the questionnaire has shown good sensitivity in identifying potential cases of oral frailty. However, it should not be considered a definitive diagnostic tool for oral frailty. Instead, it serves as a valuable resource for timely evaluations and preventive measures to maintain oral health and function in community-dwelling frail older adults. The clinical practice guidelines for oral frailty, presented by KAGD in 2022, have highlighted various intervention methods. The majority of these methods focus on preventive and non-invasive measures—such as oral care through regular dental visits, tongue strength training, and reviewing and adjusting medications—to minimize the burden on the elderly. In this context, it is more important to prioritize high sensitivity over low specificity.

Articulatory oral motor skills disability appear to play a role in the development of physical frailty and overall health status in the frail older adults [8,26,27]. The practice of repetitively articulating 3 syllables—“pa,” “ta,” and “ka”—is commonly used to assess oral motor skills, with several studies underscoring their importance. However, it is important to note that these syllables originate from the Japanese language, which may complicate their use in different cultural contexts. Consequently, the newly proposed diagnostic criteria do not include a category for oral motor skills. Despite this omission, the proposed criteria have demonstrated diagnostic validity when compared to Tanaka’s criteria, which do incorporate this parameter. Nevertheless, the importance of lip and tongue function should not be underestimated. Therefore, future diagnostic criteria should include new assessment methods for lip and tongue movement that are universally applicable to the older population.

This study has several limitations. First, the relatively small sample size may have inevitably compromised the statistical significance. Second, although various diagnostic criteria for oral frailty have been proposed recently, we applied only the criteria proposed by Tanaka. To our knowledge, Tanaka’s criteria stand out as the only set based on prospective cohort study data, despite the existence of various other criteria. Finally, due to the lack of information on physical frailty, we cannot establish an association between oral frailty, as defined by the newly developed criteria, and physical frailty. Therefore, future studies should investigate the relationship between oral and physical frailty in larger samples and using a variety of reference criteria.

The newly proposed screening questionnaire and diagnostic criteria in Korea seem to be effective tools for identifying oral frailty among community-dwelling frail older adults. This development could enhance the practicality and speed of oral frailty assessments, enabling timely and suitable interventions that may prevent both general and oral frailty, thereby contributing to increased longevity.

## Figures and Tables

**Figure f4-epih-46-e2024008:**
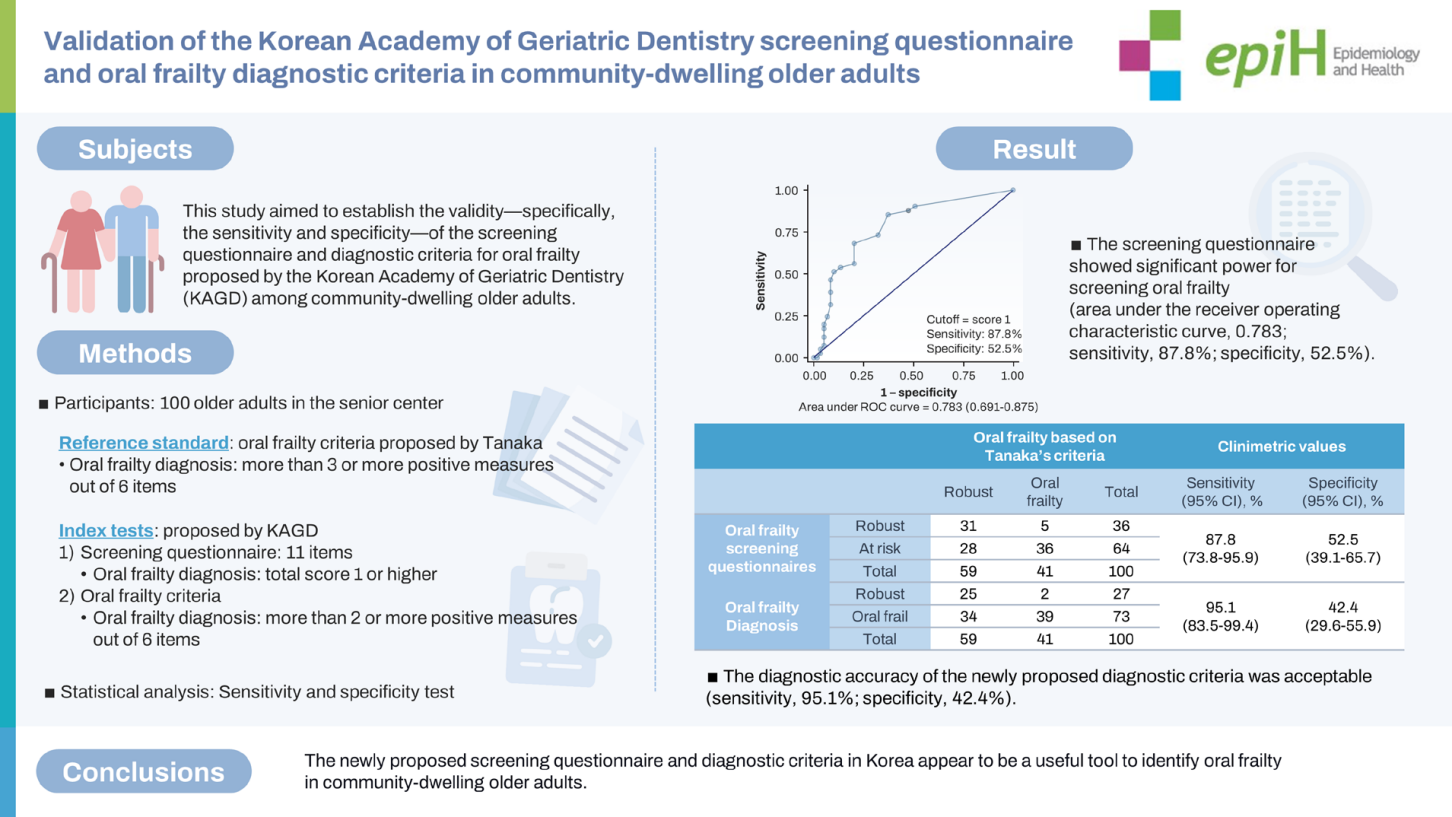


**Table 1.** Demographic characteristics

Characteristics	Total sample (n=100)	Robust (n=7)	Pre-oral frail (n=20)	Oral frail (n=73)	p-value^1^	Post-hoc
Age (yr)	78.0±7.4	71.9±4.5	74.7±7.5	79.5±7.0	0.002	Robust-Oral frail, Pre-oral frail-Oral frail
Sex (male/female)	32/68	3/4	11/9	18/55	0.029	
Body mass index (kg/m^2^)	25.1±3.5	26.2±3.0	25.1±3.2	25.0±3.7	0.704	
Comorbidities (no/yes)						
Hypertension	37/63	2/5	8/12	27/46	0.865	
Diabetes mellitus	76/24	4/3	17/3	55/18	0.321	
Heart disease	88/12	6/1	17/3	65/8	0.869	
Thyroid disorder	95/5	6/1	18/2	71/2	0.211	
Neurological disorder	94/6	7/0	19/1	68/5	0.750	
Liver disease	99/1	7/0	19/1	73/0	0.133	
Respiratory disease	99/1	7/0	20/0	72/1	0.830	
Previous cancer history	93/7	7/0	16/7	70/3	0.036	
Osteoporosis	98/2	6/1	20/0	72/1	0.051	
Hyperlipidemia	78/22	6/1	15/5	57/16	0.840	
Arthritis	87/13	7/0	19/1	61/12	0.230	
Depression	96/4	6/1	19/1	71/2	0.319	

Values are presented as mean±standard error or number.

1 From the chi-square test or independent t-test.

**Table 2.** Parameters related with oral health and function

Variables	Total sample (n=100)	Robust (n=7)	Pre-oral frail (n=20)	Oral frail (n=73)	p-value^1^	Post-hoc
No. of teeth	19.1±10.2	27.0±1.3	24.4±5.7	16.9±10.8	<0.001	Robust-Oral frail, Pre-oral frail-Oral frail
Denture wearer (no/yes)	71/29	7/0	16/4	42/32	0.017	
Chewing ability (level)^1^	4 (3-5)	4 (4-5)	5 (4-5)	4 (3-5)	0.177	
Robust/Chewing disability	86/14	7/0	20/0	59/14	0.049	
Occlusal force (n)	402.9±324.7	966.6±511.3	636.2±249.3	285.0±216.1	<0.001	Robust-Pre-oral frail, Robust-Oral frail, Pre-oral frail-Oral frail
Robust/Reduced occlusal force	31/69	7/0	15/5	9/64	<0.001	
Tongue pressure (kPa)	24.5±9.4	37.4±5.1	31.2±6.7	21.4±8.4	<0.001	Robust-Oral frail, Pre-oral frail-Oral frail
Robust/Decreased tongue pressure	30/70	7/0	13/7	10/63	<0.001	
Oral dryness	26.5±2.6	29.0±0.8	28.0±1.1	25.8±2.7	<0.001	Robust-Oral frail, Pre-oral frail-Oral frail
Robust/Oral dryness^1^	34/66	7/0	12/8	15/58	<0.001	
MWST score	5 (5-5)	5 (5-5)	5 (5-5)	5 (5-5)	0.944	
Robust/Swallowing difficulty	98/2	7/0	20/0	71/2	0.686	
Oral hygiene (score)	1 (1-1)	0 (0-1)	1 (1-1)	1 (1-1)	0.051	
Robust/Poor oral hygiene	89/11	7/0	20/0	62/11	0.102	
Oral diadochokinesis (times/sec)						
[pʌ]	4.12±1.30	4.88±0.70	4.00±1.09	4.09±1.38	0.270	
Robust/At risk^1^	12/88	2/5	1/19	9/64	0.252	
[kʌ]	4.08±1.47	5.13±1.09	4.09±1.3	3.98±1.53	0.140	
Robust/At risk^1^	15/85	3/4	3/17	9/64	0.097	
[tʌ]	4.19±1.31	5.17±1.01	4.05±1.14	4.14±1.36	0.118	
Robust/At risk^1^	15/85	3/4	3/17	9/64	0.097	
Subjective eating difficulties (no/yes)^1^	69/31	5/2	16/4	48/25	0.470	
Subjective swallowing difficulties (no/yes)^1^	92/8	7/0	20/0	65/8	0.200	

Values are presented as mean±standard error, number or median (25th-75th percentile). MWST, modified water swallowing test.

1 From the chi-square test or independent t-test.

**Table 3.** Diagnostic accuracy and associated clinimetric values of the screening questionnaire and diagnostic criteria for oral frailty

Variables		Oral frailty based on tanaka’s criteria					Clinimetric values			
		Robust (n)	Oral frailty (n)	Total (n)	Sensitivity, % (95% CI)	Specificity, % (95% CI)	LR+ (95% CI)	LR- (95% CI)	PPV, % (95% CI)	NPV,% (95% CI)
Oral frailty screening questionnaires	Robust	31	5	36	87.8 (73.8, 95.9)	52.5 (39.1, 65.7)	1.85 (1.38, 2.48)	0.23 (0.10, 0.55)	56.3 (43.3, 68.6)	86.1 (70.5, 95.3)
	At risk	28	36	64						
	Total	59	41	100						
Oral frailty diagnosis	Robust	25	2	27	95.1 (83.5, 99.4)	42.4 (29.6, 55.9)	1.65 (1.31, 2.08)	0.12 (0.03, 0.46)	53.4 (41.4, 65.2)	92.6 (75.7, 99.1)
	Oral frail	34	39	73						
	Total	59	41	100						

CI, confidence interval; LR+, positive likelihood; LR-, negative likelihood; PPV, positive predictive value; NPV, negative predictive value.

**Table 4.** Correlations between results from the screening questionnaire and diagnosis of oral frailty

Variables		Oral frailty				p-value^1^
		Robust	Pre-oral frail	Oral frail	Total	
Screening questionnaire	Robust	1	14	21	36	0.004
	At risk	4	5	25	34	
	At high risk	2	1	27	30	
	Total	7	20	73	100	

1 From chi-square test.

## Supplementary materials

Supplementary Material 1. Questionnaire for oral frailty screening

Supplementary Material 2. Measurements included in diagnostic criteria for oral frailty proposed by The Korean Academy of Geriatric Dentistry
