# Supplementary material for: Validation of the Korean Academy of Geriatric Dentistry screening questionnaire and oral frailty diagnostic criteria in community-dwelling older adults
Source: Epidemiol Health. 2023 Dec 11;46:e2024008. doi: 10.4178/epih.e2024008 (PMC11099569; doi:10.4178/epih.e2024008)
Supplement: Supplementary Material 1. — Questionnaire for oral frailty screening [file epih-46-e2024008-Supplementary-1.docx]

**Supplementary Material 1.** Questionnaire for oral frailty screening

| No | Question | Score | |
| --- | --- | --- | --- |
|  |  | Yes | No |
| 1 | Have you lost more than 3-5 kg in body weight over the past 6 months?  (※ Excluding intentional weight loss) | 3 | 0 |
| 2 | Is it difficult for you to have meals by yourself without any assistance? (related to physical frailty and cognitive impairment) | 3 | 0 |
| 3 | Is it difficult for you to brush your teeth owing to decreased general health condition compared to a year ago? | 1 | 0 |
| 4 | Do you currently feel any difficulty in eating owing to loss of teeth? | 2 | 0 |
| 5 | Do you currently experience difficulty in chewing the following foods? |  |  |
|  | - No problem in chewing |  | 0 |
|  | - Squid, steamed beef rib | 0.5 |  |
|  | - Roasted meat, kimchi | 1 |  |
|  | - Porridge, tofu | 2 |  |
| 6 | (Within the past 2 weeks) Do you need water when eating rice or dry food such as snacks? | 1 | 0 |
| 7 | (Within the past 2 weeks) Have you experienced any difficulties swallowing food? (If you have experienced, please answer the following question) |  | 0 |
|  | 7-1 Have you ever felt that food particles remain in your mouth or throat after swallowing food? | 0.5 |  |
|  | 7-2 Have you ever experienced coughing or choking while eating? | 0.5 |  |
| 8 | (Within the past 2 weeks) Have you ever noticed any changes in your voice or hoarse sounds after eating? | 1 | 0 |
| 9 | (Within the past 2 weeks) Have you frequently spilled food or liquid from your mouth while eating?  (※ Excluding occasions when you are talking during meals) | 1 | 0 |
| 10 | Is it taking significantly more time for you to eat compared to a year ago? | 1 | 0 |
| 11 | Do you feel that your speech has become somewhat slurred compared to a year ago? | 1 | 0 |
