# Supplementary material for: Validation of the Korean Academy of Geriatric Dentistry screening questionnaire and oral frailty diagnostic criteria in community-dwelling older adults
Source: Epidemiol Health. 2023 Dec 11;46:e2024008. doi: 10.4178/epih.e2024008 (PMC11099569; doi:10.4178/epih.e2024008)
Supplement: Supplementary Material 2. — Measurements included in diagnostic criteria for oral frailty proposed by The Korean Academy of Geriatric Dentistry [file epih-46-e2024008-Supplementary-2.docx]

**Supplementary Material 2.** Measurements included in diagnostic criteria for oral frailty proposed by The Korean Academy of Geriatric Dentistry

| Item | Evaluation |
| --- | --- |
| Chewing ability | The color chart value obtained from chewing color changeable gum is classified as level 1 or 2. |
| Occlusal force | The occlusal fore measured by Dental Pre-scale II® is less than 500 N. |
| Tongue pressure | The maximum tongue pressure obtained by JMS tongue pressure® is less than 30 kPa. |
| Oral dryness | The obtained value from Mucus® is less than 27.0. |
| Swallowing difficulty | The modified water swallowing test score is 3 or below. |
| Oral hygiene | An OHAT score equal to or higher than 2. |
